# A Case of Galactorrhea Associated with Excitalopram

**DOI:** 10.4306/pi.2009.6.3.230

**Published:** 2009-07-15

**Authors:** Se-Hoon Shim, Yeon-Jeong Lee, Eun-Chan Lee

**Affiliations:** 1 Department of Psychiatry, Soonchunhyang University College of Medicine, Cheonan Hospital, Cheonan, Korea.; 2 DANA Hospital, Ansan, Korea.

**Keywords:** Escitalopram, Depression, Prolactin, Galactorrhea, Side effect

## Abstract

Escitalopram is one of the most popular selective serotonin reuptake inhibitors (SSRIs) in current use as a first-line treatment for depression. Escitalopram is well-tolerated and rarely associated with serious side effects. Endocrine and reproductive side effects of serotonergic antidepressants are uncommon and galactorrhea is very rarely mentioned among SSRI-related side effects. Serotonin-enhancing antidepressants may result in a rise in prolactin levels through suppression of dopamine neurotransmission. In the present study, we report a case of hyperprolactinemic galactorrhea associated with escitalopram. A 36-year-old woman developed galactorrhea after initiation of escitalopram for depression and was found to have an elevated prolactin level. Escitalopram was discontinued with resolution of the patient's galactorrhea and normalization of her prolactin level.

## Introduction

All conventional antipsychotic drugs block D2 receptors on lactotroph cells and thus remove the main inhibitory influence on prolactin secretion.^1^,^2^ However, monoamine oxidase inhibitors, some tricyclic antidepressants, and rarely selective serotonin reuptake inhibitors (SSRIs) may increase plasma prolactin levels. With the increasing use of SSRIs, treating physicians are faced with uncommon side effects, some of which were incompletely documented at the time of the launch of these medications. Citalopram is one of the newest SSRIs, and it has a higher affinity for antiserotoninergic receptors than the other SSRIs. The pharmacological effects of citalopram are almost exclusively ascribed to the S-enantiomer, and S-citalopram (escitalopram) was recently introduced as an antidepressant. The most common side effects of escitalopram include nausea, vomiting, constipation, diarrhea, headache, sexual dysfunction, agitation, and restlessness.

Cases of hyperprolactinemia and galactorrhea induced by SSRIs including sertraline, fluoxetine, and fluvoxamine treatment were reported in some studies.^3^-^5^ However, to the best of our knowledge there are no previous reports of galactorrhea accompanied by hyperprolactinemia in Korea. In this article, we present the case of a 36-year-old woman who was treated with escitalopram for her major depressive disorder and developed galactorrhea with hyperprolactinemia that resolved upon discontinuation of the drug.

## Case

A 36-year-old woman with two children visited a psychiatric outpatient clinic on an ambulatory basis. She had no history of endocrine or reproductive pathology or psychiatric problems. Although she had been receiving a conservative treatment for iron deficiency anemia and transient hematuria, there was no specific hematologic and nephrologic finding and she had not taken any dopamine antagonistic agents including gastrointestinal motility drugs and antipsychotic agents except ferrous sulfate. Her son had been diagnosed with pervasive development disorder and mental retardation. She had suffered symptoms of depressed mood, nihilistic ideation, decreased volition, decreased psychomotor activity, loss of appetite, and sleep disturbance, which occurred after her son's diagnosis 2 years ago. Diagnostic tests were performed to ascertain whether she had neurologic or any other systemic organic disease that could explain her symptoms. Following a detailed psychiatric examination with the Structured Clinical Interview for DSM-IV Axis I Disorders (SCID-I),^6^ escitalopram 10 mg per day was prescribed to the patient with the diagnosis of major depressive disorder. She scored 26 on the Hamilton rating scale for depression (HRSD)^7^,^8^ and 25 on the Beck depression inventory (BDI).^9^,^10^ After 2 weeks of medication, her complaints declined, her HRSD score was 18 and her BDI score further decreased to 20. However, she developed galactorrhea with breast pain (the nonpuerperal discharge of milk-containing fluid from the breast). She had no history of galactorrhea. She first noticed the discharge on treatment day 14 and described it as white-creamy and from both nipples. She did not notice any bloody, greenish, or foul-smelling discharge nor report sexual dysfunction. She consulted her gynecologist, who recommended mammography and breast ultrasonography. The results of these tests were normal. However, serum prolactin level on treatment day 14 was 200 ng/mL (reference range: 1.2-24.2 ng/mL). There were no abnormalities in blood chemistry, thyroid function tests, or electrocardiogram. She was recommended to undergo magnetic resonance imaging (MRI) of the hypothalamic/pituitary area to exclude mass lesions. However, she refused the test, which was not done. Because her galactorrhea developed after initiations of her medication with escitalopram, we stopped her medication. After cessation of the medication, the discharge abated and breast pain was relieved. Her serum prolactin level decreased to 2.38 ng/mL five days after discontinuing escitalopram.

## Discussion

As the use of SSRIs increases, clinicians can expect the appearance of uncommon side effects. For example, SSRIs can induce extrapyramidal symptoms, hyperprolactinemia, galactorrhea, and mammary hypertrophy, as well as gynecomastia. On the basis of several clinical reports and neurophysiologic data about the inhibition of dopaminergic neurotransmission by SSRIs, one article^11^ proposes to classify all of these SSRI-related side effects under the term dopamine-dependent side effects.

This case demonstrates the development of hyperprolactinemia, and subsequent galactorrhea while receiving escitalopram, which subsided shortly after the discontinuation of the medication. In our approach to this patient, we sought to eliminate the most likely causes of galactorrhea. Hypothyroidism results in increased levels of thyrotropin-releasing hormone, which increases prolactin secretion. Kidneys clear prolactin, and thus, kidney disease may cause secondary hyperprolactinemia. During pregnancy, and for up to 2 years after cessation of breast-feeding, galactorrhea may be a normal finding. Because her measurements of urea, creatinine, and thyroid-stimulating hormone were all normal, and a urine human chorionic gonadotropin (HCG) test was negative, we were able to eliminate underlying kidney disease, hypothyroidism, or pregnancy as possible causes of galactorrhea. Although a transient surge (17.80±4.65 ng/mL) of prolactin following citalopram administration was found in one human study,^12^ a case with prolactin level (200 ng/mL) as extremely high as our report is very rare.

Prolactin release has been studied extensively but is not yet completely understood. The factors involved in its control include dopamine, which is potent inhibitor of prolactin release, and thyrotropin releasing hormone and serotonin (5-HT), all of which promote prolactin release.

It is not clear how serotonergic agents produce hyperprolactinemia. There is evidence that serotonin may stimulate prolactin release directly via postsynaptic 5-HT receptors in the hypothalamus,^13^ or indirectly via 5-HT mediated inhibition of tubuloinfundibular dopaminergic neurons.^14^ Clearly, the interactions between the dopaminergic and serotonergic systems throughout the brain are complex. SSRIs can inhibit dopaminergic neurotransmission not only by their effects on dopamine secretion or recapture on dopaminergic receptors, but also indirectly through serotonergic mediation.^15^ Complex changes of dopaminergic neurotransmission (mostly antidopaminergic effects) have been described with SSRIs.^16^ However, there is little synaptic contact between serotonergic fibers and dopaminergic cells, indicating that if direct inhibition of dopamine cells occurs, it is through volume transmission of serotonin in the region.^17^ There is more direct evidence for serotonergic stimulation of GABAergic neurons in the vicinity of tuberoinfundibular dopamine cells, based on the presence of 5-HT_1A_ receptors on these cells.^18^ Assuming that these GABAergic cells are interneurons, their 5-HT stimulation by SSRIs would result in inhibition of tuberoinfundibular dopamine cells, releasing the tonic inhibition of proclactin.

Gulsun et al.^19^,^20^ have previously reported two cases with escitalopram-induced galactorrhea with and without prolactinemia. As far as we know this is the first report to show the case with escitalopram-induced galactorrhea and hyperprolactinemia in Korea. While the galactorrhea sometimes seen after treatment with serotonergic medications appears to be a benign side effect, its occurrence offers new insights into the impact of serotonergic drugs on brain neurotransmitters and hormonal regulation. Clinicians should be aware of the possibility of galactorrhea in this context. Researchers should continue to explore the neurohormonal effects of serotonergic medications to gain insights into their effects.
